# Prospective comparison of prognostic scores for prediction of outcome after out-of-hospital cardiac arrest: results of the AfterROSC1 multicentric study

**DOI:** 10.1186/s13613-023-01195-w

**Published:** 2023-10-11

**Authors:** Jean Baptiste Lascarrou, Wulfran Bougouin, Jonathan Chelly, Jeremy Bourenne, Cedric Daubin, Olivier Lesieur, Pierre Asfar, Gwenhael Colin, Marine Paul, Nicolas Chudeau, Gregoire Muller, Guillaume Geri, Sophier Jacquier, Nicolas Pichon, Thomas Klein, Bertrand Sauneuf, Kada Klouche, Martin Cour, Caroline Sejourne, Filippo Annoni, Jean-Herle Raphalen, Arnaud Galbois, Cedric Bruel, Nicolas Mongardon, Nadia Aissaoui, Nicolas Deye, Julien Maizel, Florence Dumas, Stephane Legriel, Alain Cariou, the AfterROSC Network, Noémie Peres, Audrey Le Saux, Mathieu Bellal, Maxime Leloup, Paul Jaubert, Matthieu Henry-Lagarrigue, Nina Alezra, Juliette Meunier, Mai-Anh Nay, Charlotte Salmon-Gandonnière, Sandrine Mons, Bruno Levy, Xavier Souloy, Laura Platon, Laurent Argaud, Fabio Taccone, Damien Vimpere, Riad Chelha, Quentin de Roux, Caroline Hauw Berlemont, Bruno Megarbane, Sarah Benghanem, Jeremie Lemarie, Cyril Goulenok

**Affiliations:** 1 AfterROSC Network Group, Paris, France; 2 Université de Paris Cité, Inserm, Paris Cardiovascular Research Center, Paris, France; 3 Service de Médecine Intensive Réanimation, University Hospital Center, 30 Boulevard Jean Monet, 44093 Nantes Cedex 9, France; 4 Médecine Intensive Réanimation, Hôpital Jacques Cartier, Massy, France; 5 Médecine Intensive Réanimation, CH Toulon, Toulon, France; 6 Réanimation des Urgences et Déchocage, CHU La Timone, APHM, Marseille, France; 7 Médecine Intensive Réanimation, CHU Caen, Caen, France; 8 Médecine Intensive Réanimation, CH La Rochelle, La Rochelle, France; 9 Médecine Intensive Réanimation, CHU Angers, Angers, France; 10 Médecine Intensive Réanimation, CHD Vendée, La Roche-Sur-Yon, France; 11 Médecine Intensive Réanimation, CH Versailles, Le Chesnay, France; 12 Médecine Intensive Réanimation, CH Le Mans, Le Mans, France; 13 Médecine Intensive Réanimation, CHR Orléans, Orléans, France; 14 Médecine Intensive Réanimation, APHP, CHU Ambroise Pare, Boulogne-Billancourt, France; 15 Médecine Intensive Réanimation, CHU Tours, Tours, France; 16 Médecine Intensive Réanimation, CH Brive-La-Gaillard, Bourges, France; 17 Médecine Intensive Réanimation, CHU Nancy, Nancy, France; 18 Médecine Intensive Réanimation, CH Cherbourg-en-Cotentin, Cherbourg, France; 19 Médecine Intensive Réanimation, CHU Montpellier, Montpellier, France; 20 Médecine Intensive Réanimation, Hospices Civils Lyon, Lyon, France; 21 Médecine Intensive Réanimation, CH Bethune, Bethune, France; 22 Réanimation, ERASME, Brussels, Belgium; 23 Médecine Intensive Réanimation, APHP, CHU Necker, Paris, France; 24 Service de Réanimation Polyvalente, Hôpital Privé Claude Galien, Quincy-Sous-Sénart, France; 25 Service de Réanimation Polyvalente, Groupe Hospitalier Paris Saint Joseph, Paris, France; 26 Service d’Anesthésie-Réanimation Chirurgicale, APHP, CHU Henri Mondor, Créteil, France; 27 Médecine Intensive Réanimation, APHP, HEGP, Paris, France; 28 Médecine Intensive Réanimation, APHP, CHU Lariboisière, Paris, France; 29 Médecine Intensive Réanimation, CHU Amiens, Amiens, France; 30 Service Des Urgences, APHP, CHU Cochin, Paris, France; 31 Médecine Intensive Réanimation, APHP, CHU Cochin, Paris, France

**Keywords:** Cardiac arrest, Outcome prediction, Neurological prognosis, Functional outcome, Score

## Abstract

**Background:**

Out-of-hospital cardiac arrest (OHCA) is a heterogeneous entity with multiple origins and prognoses. An early, reliable assessment of the prognosis is useful to adapt therapeutic strategy, tailor intensity of care, and inform relatives. We aimed primarily to undertake a prospective multicentric study to evaluate predictive performance of the Cardiac Arrest Prognosis (CAHP) Score as compare to historical dataset systematically collected after OHCA (Utstein style criteria). Our secondary aim was to evaluate other dedicated scores for predicting outcome after OHCA and to compare them to Utstein style criteria.

**Methods:**

We prospectively collected data from 24 French and Belgium Intensive Care Units (ICUs) between August 2020 and June 2022. All cases of non-traumatic OHCA (cardiac and non-cardiac causes) patients with stable return of spontaneous circulation (ROSC) and comatose at ICU admission (defined by Glasgow coma score ≤ 8) on ICU admission were included. The primary outcome was the modified Rankin scale (mRS) at day 90 after cardiac arrest, assessed by phone interviews. A wide range of developed scores (CAHP, OHCA, CREST, C-Graph, TTM, CAST, NULL-PLEASE, and MIRACLE2) were included, and their accuracies in predicting poor outcome at 90 days after OHCA (defined as mRS ≥ 4) were determined using the area under the receiving operating characteristic curve (AUROC) and the calibration belt.

**Results:**

During the study period, 907 patients were screened, and 658 were included in the study. Patients were predominantly male (72%), with a mean age of 61 ± 15, most having collapsed from a supposed cardiac cause (64%). The mortality rate at day 90 was 63% and unfavorable neurological outcomes were observed in 66%. The performance (AUROC) of Utstein criteria for poor outcome prediction was moderate at 0.79 [0.76–0.83], whereas AUROCs from other scores varied from 0.79 [0.75–0.83] to 0.88 [0.86–0.91]. For each score, the proportion of patients for whom individual values could not be calculated varied from 1.4% to 17.4%.

**Conclusions:**

In patients admitted to ICUs after a successfully resuscitated OHCA, most of the scores available for the evaluation of the subsequent prognosis are more efficient than the usual Utstein criteria but calibration is unacceptable for some of them. Our results show that some scores (CAHP, sCAHP, mCAHP, OHCA, rCAST) have superior performance, and that their ease and speed of determination should encourage their use.

*Trial registration* https://clinicaltrials.gov/ct2/show/NCT04167891

**Supplementary Information:**

The online version contains supplementary material available at 10.1186/s13613-023-01195-w.

## Background

In Europe, more than 300,000 out-of-hospital cardiac arrests (OHCAs) occur each year, resulting in 250,000 deaths [1]. Among these, less than 10% will leave the hospital alive without serious neurological sequelae [2]. Nearly all deaths occur early, during the first days and weeks, as a consequence of lesions caused by hypoxic-ischemic brain injury, which are aggravated by reperfusion provoked by the return of spontaneous circulation (ROSC). Clinicians, who face many challenges, find it very useful to be able to estimate the subsequent prognosis with maximum reliability so that they can give relatives reliable information and adapt the therapeutic strategy. In addition, an estimation of the accuracy of the prognosis may help to better identify subgroups of patients eligible for certain interventions and clinical research programs, such as early coronary reperfusion [3] or neuroprotective treatments [4].

To allow an early assessment of prognosis, it is possible to use scores based on variables available immediately upon admission to the intensive care unit (ICU). Severity scores used in the general population of ICU patients have been evaluated in the specific population of OHCA patients, but revealed poor calibration and discrimination performances [5–9]. On the other hand, numerous specific scores, all available at hospital admission, have currently been developed within the restricted framework of OHCA patients [9–19]. For example, the Cardiac Arrest Hospital Prognosis (CAHP) score was evaluated in this situation and showed acceptable discrimination and calibration performance [10, 11]. However, to our knowledge, the respective performances of all these scores have never been compared simultaneously for the same population of patients in a prospective multicenter study. Historically, Utstein criteria (which are also used in the calculation of all these scores) were used for prognostication after OHCA at the prehospital phase, and recommendations were made to collect these elements under the term “Utstein style” [20–22]. Thus, as these Utstein criteria are the minimum framework to be collected in a study on OHCA, it seemed important to analyze the respective discrimination of each score as compared to the historical reference Utstein variable-based model score.

Thus, we designed the AfterROSC1 study with the main objective of comparing the performance of the CAHP score compared to the score derived from the Utstein style criteria for the prediction of functional prognosis after cardiac arrest. The secondary objectives were to compare the performance of other scores specific to cardiac arrest to those of the score derived from the Utstein style criteria.

## Methods

The main objective of the present research was to evaluate, in a prospective, multicenter, observational study, the discrimination, calibration, and clinical utility of CAHP score after OHCA as compared to Utstein criteria. The secondary objective was to compare the performance of these different scores to the prediction offered by the Utstein criteria. The study was declared on ClinicalTrial.gov before it began (NCT04167891; August 1, 2020). Statistical analysis plan was approved before enrolment of first patient by Ethics Committee in charge of the study in France.

### Study settings

This study was conducted in 24 ICUs in France and Belgium between August 2020 and June 2022. All were members of the AfterROSC Network, which is dedicated to the promotion and development of clinical research and education regarding post-cardiac arrest care.

### Ethics

Information about the study was delivered to each patient’s relatives. In cases of missing relatives, emergency inclusion was allowed according to French law. Patients without an available relative included in the study were informed as soon as they regained competence. If they subsequently declined to participate, they were removed from the database. The research protocol (available with the full text of this article) was approved by the appropriate ethical committees (2019-A01378-49; CPP-SMIV 190901) and French data-protection authorities, according to the principles of the Declaration of Helsinki and its amendments. The analysis and reporting for this study were conducted in accordance with the Transparent Reporting of a Multivariable Prediction Model for Individual Prognosis or Diagnosis (TRIPOD) statement [14].

### Study population

All patients admitted to the participating centers during the study period were screened for participation. Patients were eligible if they were 18 years or older, if they were admitted to the ICU after OHCA, and if they remained comatose at admission (defined by Glasgow Coma Score equal to or lower than 8) despite ROSC. In patients who had been sedated before ICU admission, the Glasgow Coma Score (GCS) determined by the emergency physician just before sedation was used. Non-inclusion criteria were in-hospital cardiac arrest, traumatic cardiac arrest, patient under guardianship, and previous inclusion in the AfterROSC1 study. Furthermore, we only included in the analysis patients for whom all the Utstein style criteria and the main endpoint (modified Rankin scale on day 90) were available.

#### Score determination

For each patient, all components of the Utstein style criteria were captured and plotted into a dedicated score [15]. According to reference [15] and to a previous study with the same methodology [13], age, gender, cardiac/non cardiac cause of arrest, bystander, bystander cardiopulmonary resuscitation (CPR), location (home/other), occurrence of CA before Emergency Medical System (EMS) arrival, shockable/non shockable rhythm, and time between CA and EMS arrival were incorporated into the “Utstein style” score.

Similarly, for each patient, individual scores were calculated according to published data. To select scores retained in the present analysis, we performed a narrative review of observational studies published from database inception (1947) until September 2019 that included non-traumatic OHCA patients. We included studies that reported both early prognostic scores (including prehospital and in-hospital variables) and patient outcomes, which included early mortality (within 24 h after emergency department admission), survival to hospital admission, survival to hospital discharge, and functional outcome at hospital discharge.

The following databases were searched: PubMed, Embase, Google Scholar, and Web of Science. The search strategies, adapted for each database, included medical subject headings and keywords for “heart arrest, ventricular fibrillation, resuscitation, pulseless electrical activity, asystole” combined using the Boolean operator AND with a comprehensive range of search terms for prognostic score, including “score, early determination of prognosis.”

All risk scores calculable upon admission to hospital and predicting patients’ outcome after OHCA were retained in the analysis. Eight scores were selected: CAHP [9] and its simplified [10] and modified versions [11], OHCA [11], CREST [12], C-Graph [14], TTM [15], CAST [16], and NULL-PLEASE [17]. Post hoc, we added two scores, rCAST [16] and MIRACLE2 [17], published during the study period. To facilitate comparison, the variables involved in the calculation of these scores are listed in Additional file 1: Table S1.

### Data collection

All data were collected by a dedicated study nurse or investigator in each participating center. The following variables were collected: baseline clinical data and comorbidities; characteristics of cardiac arrest and resuscitation; clinical and biological characteristics at ICU admission; treatments delivered in the ICU; length of stay (LOS) in ICU; invasive mechanical ventilation duration; functional and vital status at ICU discharge; and functional and vital status at hospital discharge. Post-resuscitation shock (PRS) was recorded at ICU admission and was defined as a systolic blood pressure below 90 mm Hg for at least 30 min with impaired end-organ perfusion (cool extremities, mottling, or urine output < 30 mL/h), requiring norepinephrine and/or epinephrine intravenous infusion [18]. The last neurological evaluation was performed on day 90 using the modified Rankin scale [19].

### Outcome measures

The neurological outcome was scored using the level reached on the modified Rankin scale [20] at day 90, assessed by a research nurse during a telephonic interview. The main endpoint was a favorable outcome at Day 90, as defined by an mRS level of 0 (no symptoms), 1 (no significant disability), 2 (slight disability) or 3 (moderate disability), as recommended in the guidelines [21].

### Sample size

Using existing data from a large and comprehensive registry of cardiac arrests admitted to the intensive care unit in the Greater Paris area [2], according to the same inclusion criteria of the study described here, multivariable logistic regression integrating the Utstein criteria described allows the realization of a receiver operating characteristic (ROC) curve whose area is estimated at 0.85 [13]. The prediction of the CAHP score has been described as having an area under the curve of 0.93 [10]. Considering a first-species risk of 0.05, a power of 0.90, and a difference of 0.85 to 0.93, it was necessary to include 574 patients. According to previous data, we planned a favorable functional outcome rate of 20% for patients included at ICU admission [2]. Since the endpoint was based on a telephonic interview at 90 days and considering the risk of about 20% of missing responses on the modified Rankin endpoint at 90 days, a total of 597 patients were required [22].

### Statistical analysis

We used descriptive statistics to summarize categorical variables as proportions, and continuous variables as mean with standard deviation and median with interquartile range for normal and non-normal distribution, respectively. Comparisons between proportions used Pearson’s chi-squared (or Fisher’s exact test, if appropriate) and a *t*-test (or Mann–Wilcoxon rank-sum test) for continuous variables.

The discrimination abilities of the prognostication scores were assessed using ROC analysis and quantified using the area under the ROC curve (AUC). The AUC values were compared in a pairwise manner using the method of DeLong et al. [23]. The calibration performances of the prognostication scores were assessed using the Hosmer–Lemeshow test. For complete assessment of calibration and regarding low power of Hosmer–Lemeshow test, we performed calibration belt—which plot expected and observed outcome according to each score—with related P-value using calibration belt function on STATA. In the absence of dedicated metric for balance between discrimination ability and simplicity for determination, we added the ratio between AUC and number of items for each score. We plotted a decision curve analysis for Utstein style criteria score and others scores [24].

A first sensitivity analysis was performed to determine AUROC of each score including missing data, with performed multiple imputations using a chained equations [25] on the dataset restricted to patients with available day-90 functional outcome available (primary outcome), and based on *M* = 10 imputed completed. A second sensitivity analysis was performed, restricted to non-cardiac causes of cardiac arrest at ICU admission.

All tests were two-sided, with a *P*-value of < 0.05 considered significant. Analyses were performed using STATA/SE 14.2 (Lakeway Drive, TX, USA).

## Results

### Baseline characteristics

**Fig. 1:**
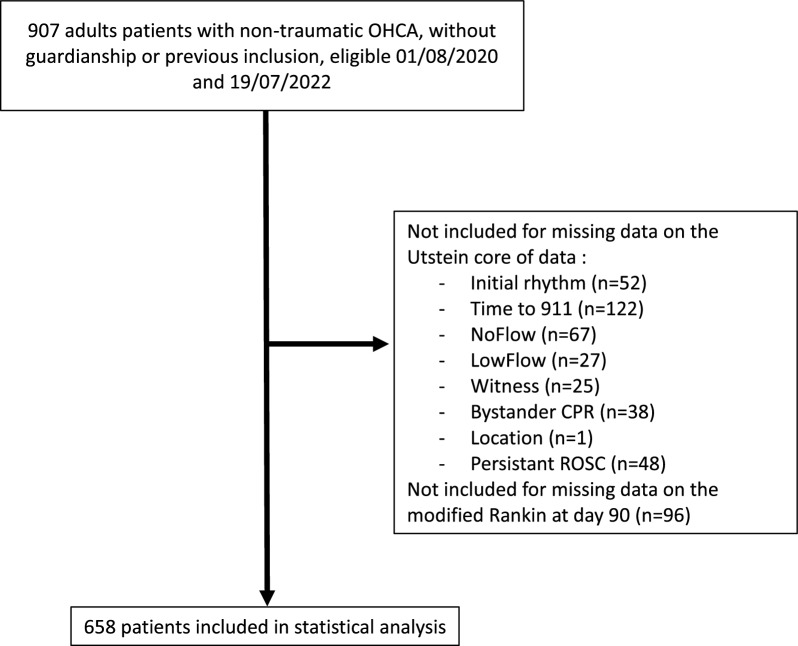
Study flowchart

During the study period, 907 patients were screened for participation, and 658 were retained in the analysis (Fig. 1). Baseline characteristics and outcomes are described in Table 1. Patients were mostly male (72%) and collapsed at home (64%) in the presence of a witness (86%) who performed bystander CPR in 68% of cases.

**Table 1:** Characteristics of the study population

	Missing	All (*N* = 658)
Male, *n*, %	0	478 (72%)
Age, ± SD	0	61 ± 15
Charlson comorbidities index, median [IQR]	0	2 [1–4]
Home location, *n*, %	0	423 (64%)
Witness, *n*, %	0	566 (86%)
Bystander CPR, *n*, %	0	449 (68%)
Occurrence of cardiac arrest after EMS arrival, *n*, %	0	88 (13%)
Shockable rhythm, *n*, %	0	318 (48%)
No flow duration, min, median [IQR]	0	3 [0–8]
Low flow duration, min, median [IQR]	0	20 [15–30]
Time to 911, min, median [IQR]	0	10 [5–15]
Epinephrine use, *n*, %	0	491 (75%)
Epinephrine dose, mg, median [IQR]	0	2 [0–4]
First arterial pH, ± SD	9	7.26 [7.14–7.35]
Post resuscitation shock, *n*, %	1	355 (54%)
ST elevation, *n*, %	3	225 (34%)
Early invasive coronary strategy, *n*, %	26	351 (56%)
Cardiac cause as supposed origin of arrest, *n*, %	3	422 (64%)
Targeted temperature management between 32° to 36 °C, *n*, %	0	568 (86%)
Survival at ICU discharge, *n*, %	0	249 (38%)
Survival at day 90, *n*, %	0	241 (37%)
Favorable functional outcome at day 90, *n*, % mRS 3–2–1–0	0	223 (34%)

CPR: Cardiopulmonary resuscitation; ICU: Intensive care unit; IQR: Interquartile range; SD: Standard deviation; mRS: modified Rankin Scale

### Functional outcome and mortality

**Fig. 2:**
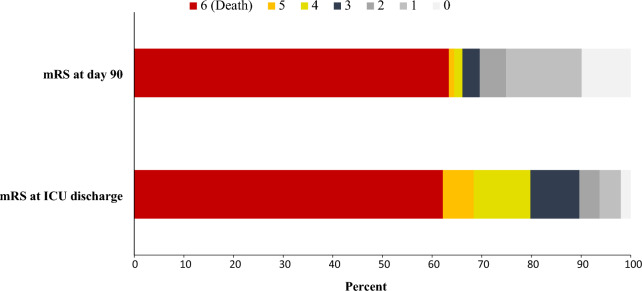
Distribution of mRS scores in each category at ICU discharge and day 90 follow-up

Survival at ICU discharge and at day 90 was 38%, with a favorable functional outcome (mRS < 4) at day 90 observed in 37% of cases (Fig. 2).

### Discrimination, calibration and comparison of CAHP score to “Utstein style criteria score” (Table 2)

**Table 2:** Comparison (total sample size *N* = 658)

	Utstein	CAHP	sCAHP	mCAHP	OHCA	CREST	C-GRApH	TTM	NULL-PLEASE	rCAST	MIRACLE2
Number of items for score determination	8	7	6	6	5	5	5	10	9	5	7
Number of patients with calculated score	658	649	649	649	640	544	607	557	640	625	620
Proportion of patients with score available as compared to full cohort	100.0%	98.6%	98.6%	98.6%	97.3%	82.6%	92.2%	84.6%	97.3%	94.9%	94.2%
Median score, IQR	NA	147 [111–175]	132 [101–156]	94 [73–110]	28 [13–41]	2 [1–3]	2 [1–3]	17 [12–20]	5 [3–6]	9 [6.5–12]	4 [2–5]
AUROC (95% CI)	0.79 [0.76–0.83]	0.87 [0.84–0.90]	0.85 [0.81–0.87]	0.86 [0.83–0.89]	0.84 [0.81–0.88]	0.79 [0.75–0.83]	0.76 [0.71–0.80]	0.88 [0.86–0.91]	0.81 [0.77–0.84]	0.82 [0.78–0.85]	0.85 [0.82–0.88]
Hosmer–Lemeshow											
Absolute value	5.48	11.03	4.83	7.63	11.92	8.84	4.43	12.14	3.84	9.28	16.64
*P*-value	0.70	0.19	0.77	0.47	0.15	0.26	0.48	0.14	0.87	0.32	0.03
Comparison versus Utstein Delta AUROC (95% CI)	NA	0.08 [0.07–0.08]	0.05 [0.04–0.06]	0.07 [0.06–0.07]	0.05 [0.05–0.05]	0 [0–0]	− 0.03 [− 0.03 to 0.05]	0.09 [0.08–0.10]	0.01 [0.01–0.02]	0.02 [0.02–0.03]	0.06 [0.05–0.06]
P-value		< 0.0001	< 0.001	< 0.001	< 0.001	0.28	0.03	< 0.001	0.20	0.16	< 0.001
Added AUROC per item	0.09	0.12	0.14	0.14	0.17	0.16	0.15	0.08	0.09	0.16	0.12

NA: Not applicable. AUROC: Area under the ROC curve. IQR: interquartile range

CAHP score could be determined for 98.6% of patients. The AUROC for CAHP was 0.87 [0.84–0.90] which was significantly higher as compare to reference (0.79 [0.76–0.83]; *P* < 0.001). The calibration was acceptable according to Hosmer–Lemeshow test and *calibration belt* test (both *P*-value > 0.05).

### Discrimination, calibration and comparison of other scores to “Utstein style criteria score” (Table 2)

The proportion of patients for whom it was possible to calculate each of the scores studied varied between 82.6% (CREST) and 98.6% (sCAHP, and mCAHP). According to Hosmer–Lemeshow test, calibration was acceptable for all score except for MIRACLE2 (*P* = 0.03).

**Fig. 3:**
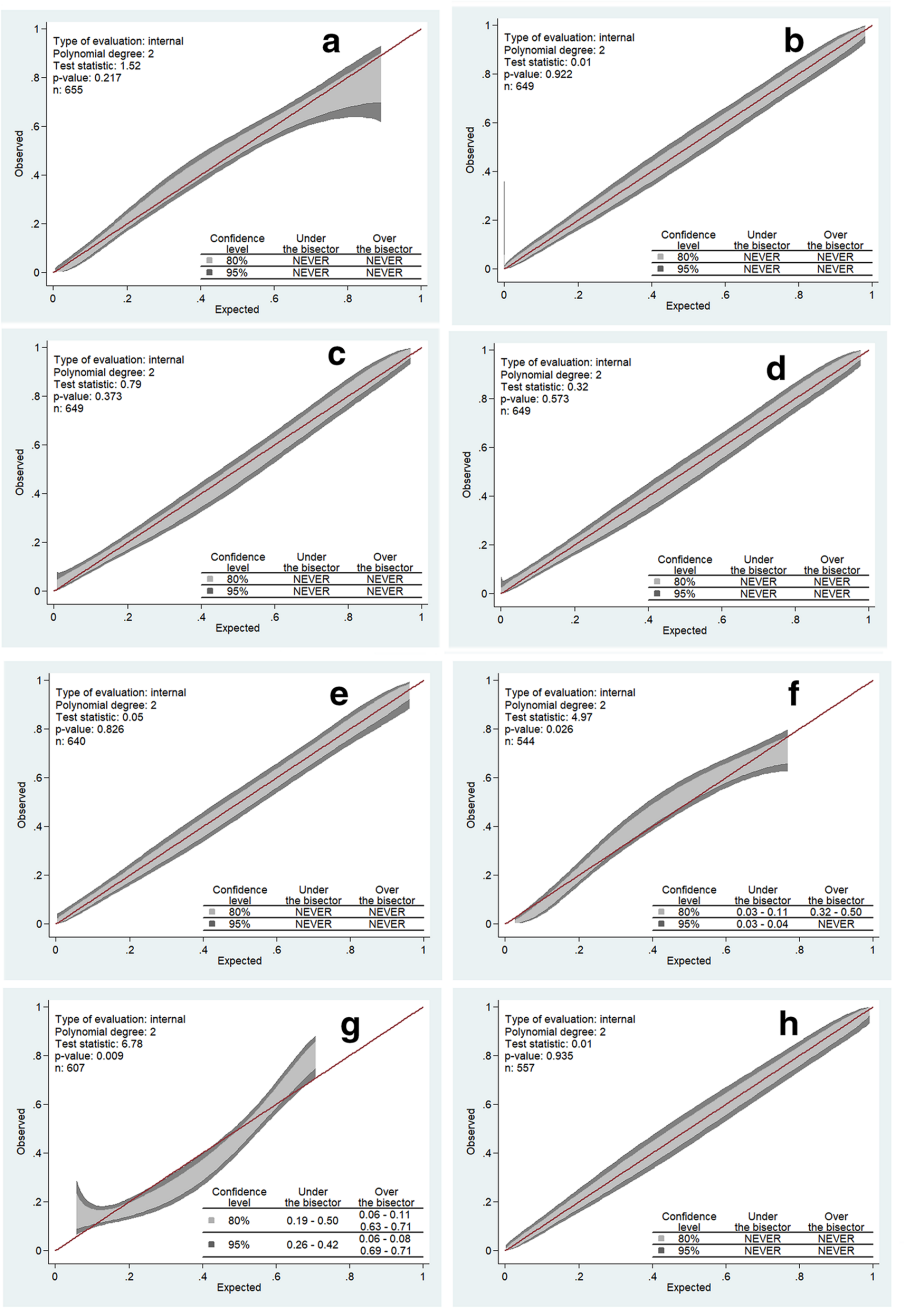

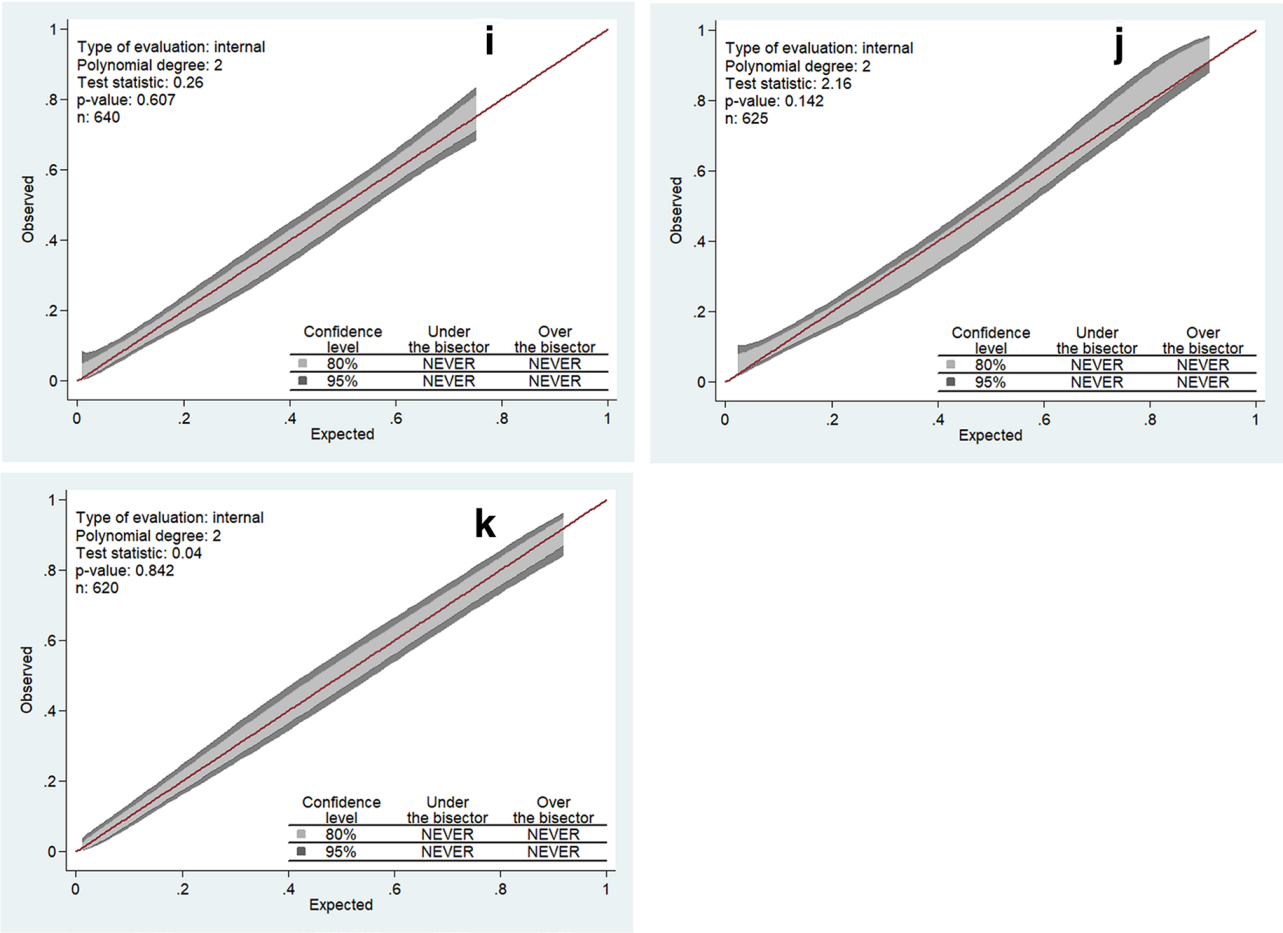
Calibrations belts

According to calibration belt test, calibration was acceptable for all score except for CREST and CGRAPH (*P*-values, respectively, 0.02 and 0.01). Calibration belts are depicted in Fig. 3.

**Fig. 4:**
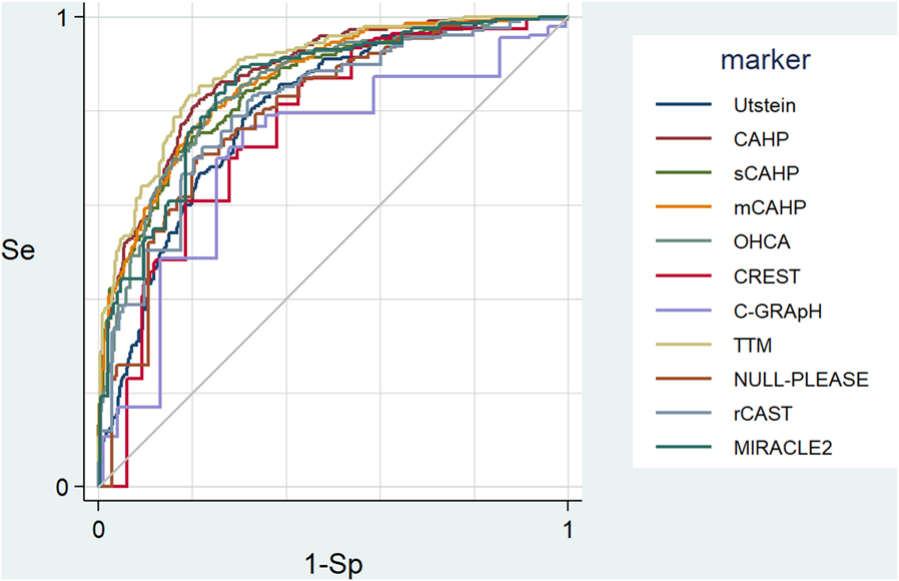
ROC curves of scores included in the analysis

Comparing AUROCs, the three best-performing scores were achieved by TTM (0.88 [0.86–0.92]), CAHP (0.87 [0.84–0.90], and mCAHP (0.86 [0.83–0.89]), while the three worst-performing scores were achieved by C-GRAPH (0.76 [0.71–0.80]), CREST (0.79 [0.75–0.83]), and NULL-PLEASE (0.81 [0.77–0.84]). A comparison of the respective AUROCs is depicted in Fig. 4. All scores showed significantly increased AUROC values (*P* < 0.05) in comparison with the Utstein style “score” except for CREST (*P* = 0.28), NULL-PLEASE (*P* = 0.20) and rCAST (*P* = 0.16). For each score, the added value of each component (total AUROC/number of items) appears in Table 2. AUROC values for each score after multiple imputation are available on Additional file 2: Table S2.

### Clinical utility

Decision curve analysis is available as Additional file 4: Fig. S1.

### Performances in patients with a non-cardiac cause of arrest (Additional file 3: Table S3)

In the subgroup of patients with a non-cardiac cause of arrest (*n* = 233), the AUROC of the Utstein style score was 0.75 [0.67–0.83]. The predictive values of CREST and NULL-PLEASE could not be determined because these scores are not usable in this population. AUROCs from other scores ranged from 0.59 [0.48–0.70] to 0.87 [0.81–0.93]. The scores for CAHP, mCAHP, and TTM performed significantly better than Utstein, whereas C-GRAPH performed significantly worse.

## Discussion

In this prospective multicenter study, we found that most of the tested predictive scores performed at least as well as, and most often better than, the predictive score derived from the Utstein style. In the population studied, we observed that these predictive scores could be calculated on admission in nearly all patients, confirming that they could be used routinely.

These results should be considered in relation to the data available in this field. Isenschmid et al*.* [26] found that prediction scores dedicated to cardiac arrest cohorts performed better than general ICU scores and that the presumed asphyxia cause of cardiac arrest was associated with a drop in AUROC (0.71 vs 0.83). Potpara et al*.* [27], monitoring a cohort of 547 patients who suffered from OHCA, observed that the NULL-PLEASE needed to be modified regarding pH and lactate values, as those two items were inconsistently measured in their cohort. Tsuchida et al*.* found in a cohort of 236 OHCA patients that AUROC of NULL-PLEASE, CAST, and rCAST were 0.874, 0.860, and 0.770, respectively [28]. Recently, Blatter et al*.* [29], observing 415 patients, found that the AUROCs of OHCA, CAHP, APACHE II, and SAPS II scores had similar performances in predicting poor neurological outcomes at 2 years after cardiac arrest. Note that “general ICU scores,” such as APACHE II and SAPS II, could only be determined after 24 h, a limitation of their utility in the early phase of evolution. Heo et al*.* [30] compared 12 scores in a dataset of 1163 patients suffering from OHCA. The PROLOGUE score showed better discrimination performance without miscalibration. However, their study included a relatively homogenous population of patients who received targeted temperature management, and the analysis was retrospective, with only 69% of the population eligible for score determination. To summarize, the literature is extensive but has recurrent limitations (mostly retrospective design and small sample size). A common limitation is also the endpoint timing, with some of these studies using a short-term evaluation (ICU discharge) and others using a long-term evaluation (up to 2 years after the index event), which is questionable. Following the guidelines of the last version of the Core Outcome Set for Cardiac Arrest [21], we used the mRS, which allows for a combined assessment of neurological outcome and vital status at day 90. Hosmer–Lemeshow test was significant for MIRACLE2 indicating mis-calibration for this score. Calibration was not adequate for CREST and C-Graph. TTM score could not be determined for more than 10% of patients. In the subgroup of non-cardiac OHCA, CREST and NULL-PLEASE could not be determined. All those together, leave CAHP (and its subsequent scores), OHCA, NULL-PLEASE and rCAST as candidates for universal adoption. 

Early evaluation of patients’ prognoses at hospital or ICU admission is very useful to tailor interventions, especially neuroprotective interventions. However, it is likely too few clinicians make the assessment. Scores are good candidates for this assessment provided they have been scientifically validated. On other side, besides AUCs values, prognostic tests with similar discrimination power could mask different clinical utilities: scores with very high specificity can be useful for accurate identification (rule in) specific outcome, whereas scores with very high sensitivity can be useful for exclusion (rule out) of specific outcome. However, our sample size did not allow us determination of respective characteristics of each score for predetermined Specificity. Some retrospective studies have already found than scores could help in identification of subgroups of interest (such as for coronary angiogram [3] or temperature management [11]) and furthers interventional trials could use them as selection criterions. Their use could be encouraged by guidelines to promote their use by clinicians on a daily basis. As already highlighted, recent literature has evaluated composite scores with many limitations. Apart from prediction scores, other tools could be used. Deye et al*.* [31] found that early determination of PS100-B have acceptable AUROCs (AUC 0.83 [95% CI 0.78–0.88]), but this biomarker is rarely available on an emergency basis, which makes its use impossible when a decision must be made without delay. Other brain damage biomarkers exist that are potentially interesting in predicting outcomes in these patients, such as serum neurofilament light (NFL) [32] and Tau protein. However, their half-lives are too long for their use in this early phase, and preliminary data have indicated low performance (AUROC 0.58 [0.48–0.69] for tau protein [33]). A quantitative evaluation of pupillary light reflex may also be a valuable tool, but it is not widely available at this time [34].

To be effective, a risk score needs to be accurate, well calibrated, and easy to employ in routine practice. Whereas several metrics exist to measure usability in commercial areas, there is no equivalent for medical scores. Indeed, effectiveness is defined by discrimination and calibration, whereas efficiency and satisfaction cannot be determined with already validated tools. To help readers, we chose to determine the ratio of added AUROC per item included in the score to reflect the added value of each item in the score. However, this metric did not consider completeness or ease of determination for each value.

Our study has several strengths. First, it is the largest study to date to evaluate prognosis scores. Second, we compare a very wide range of these scores in a unique, prospective, and dedicated study. Third, we highlight than prognosis can be performed relatively easily at the bedside.

Our study must be interpreted within its own limits. We did not evaluate all the available scores because we did not capture the data required for their determination [35–37] or because they were developed in a specific context of care [38–40]. Finally, some scores included dynamic values, such as for a vasopressor dose [41]. Our sample size, although larger than that used in previous studies, could lack the power to detect small differences, leading to recruitment in AfterROSC2 (NCT05606809).

## Conclusion

In patients admitted to intensive care after a cardiac arrest, most of the scores available for evaluation of the subsequent prognosis are more efficient than the usual Utstein criteria. Some of these scores performed better than others, but calibration is unacceptable for some of them. Our results show that some scores (CAHP, sCAHP, mCAHP, OHCA, rCAST) have superior performance, and that their ease and speed of determination should encourage their use.

### Supplementary Information

**Additional file 1: Table S1.** Description of each score.

**Additional file 2: Table S2.** Comparison of AUC after multiple imputations.

**Additional file 3: Table S3.** Comparison of scores in the restricted population of patients with cardiac arrests from non-cardiac causes.

**Additional file 4: Figure S1.** Decision curve.

## Data Availability

Data are available on reasonable request to the first author and after approval by the steering committee of the AfterROSC Network.

## References

[1] Survival after out-of-hospital cardiac arrest in Europe - Results of the EuReCa TWO study - Resuscitation. https://www.resuscitationjournal.com/article/S0300-9572(20)30046-0/fulltext. Accessed 28 Sep 2021 10.1016/j.resuscitation.2019.12.042 32027980

[2] Bougouin W, Lamhaut L, Marijon E (2014). Characteristics and prognosis of sudden cardiac death in Greater Paris: population-based approach from the Paris Sudden Death Expertise Center (Paris-SDEC). Intensive Care Med.

[3] Bougouin W, Dumas F, Karam N (2018). Should we perform an immediate coronary angiogram in all patients after cardiac arrest?: insights from a large French Registry. JACC Cardiovasc Interv.

[4] Holgersson J, Meyer MAS, Dankiewicz J (2022). Hypothermic versus normothermic temperature control after cardiac arrest. NEJM Evid.

[5] Choi JY, Jang JH, Lim YS (2018). Performance on the APACHE II, SAPS II, SOFA and the OHCA score of post-cardiac arrest patients treated with therapeutic hypothermia. PLoS ONE.

[6] Donnino MW, Salciccioli JD, Dejam A (2013). APACHE II scoring to predict outcome in post-cardiac arrest. Resuscitation.

[7] Bisbal M, Jouve E, Papazian L (2014). Effectiveness of SAPS III to predict hospital mortality for post-cardiac arrest patients. Resuscitation.

[8] Niskanen M, Kari A, Nikki P (1991). Acute physiology and chronic health evaluation (APACHE II) and Glasgow coma scores as predictors of outcome from intensive care after cardiac arrest. Crit Care Med.

[9] Salciccioli JD, Cristia C, Chase M (2012). Performance of SAPS II and SAPS III scores in post-cardiac arrest. Minerva Anestesiol.

[10] Maupain C, Bougouin W, Lamhaut L (2016). The CAHP (Cardiac Arrest Hospital Prognosis) score: a tool for risk stratification after out-of-hospital cardiac arrest. Eur Heart J.

[11] Lascarrou JB, Dumas F, Bougouin W (2022). Differential effect of targeted temperature management between 32°C and 36°C following cardiac arrest according to initial severity of illness: insights from two international data sets. Chest.

[12] Dyson K, Brown SP, May S (2019). International variation in survival after out-of-hospital cardiac arrest: a validation study of the Utstein template. Resuscitation.

[13] Rea TD, Cook AJ, Stiell IG (2010). Predicting survival after out-of-hospital cardiac arrest: role of the Utstein data elements. Ann Emerg Med.

[14] Collins GS, Reitsma JB, Altman DG, Moons KGM (2015). Transparent reporting of a multivariable prediction model for individual prognosis or diagnosis (TRIPOD). Circulation.

[15] Perkins GD, Jacobs IG, Nadkarni VM (2015). Cardiac arrest and cardiopulmonary resuscitation outcome reports: update of the Utstein Resuscitation Registry Templates for Out-of-Hospital Cardiac Arrest: a statement for healthcare professionals from a task force of the International Liaison Committee on Resuscitation (American Heart Association, European Resuscitation Council, Australian and New Zealand Council on Resuscitation, Heart and Stroke Foundation of Canada, InterAmerican Heart Foundation, Resuscitation Council of Southern Africa, Resuscitation Council of Asia); and the American Heart Association Emergency Cardiovascular Care Committee and the Council on Cardiopulmonary, Critical Care, Perioperative and Resuscitation. Circulation.

[16] Nishikimi M, Ogura T, Nishida K (2019). External validation of a risk classification at the emergency department of post-cardiac arrest syndrome patients undergoing targeted temperature management. Resuscitation.

[17] Pareek N, Kordis P, Beckley-Hoelscher N (2020). A practical risk score for early prediction of neurological outcome after out-of-hospital cardiac arrest: MIRACLE2. Eur Heart J.

[18] Ziriat I, Le Thuaut A, Colin G (2022). Outcomes of mild-to-moderate postresuscitation shock after non-shockable cardiac arrest and association with temperature management: a post hoc analysis of HYPERION trial data. Ann Intensive Care.

[19] Bonita R, Beaglehole R (1988). Recovery of motor function after stroke. Stroke.

[20] van Swieten JC, Koudstaal PJ, Visser MC (1988). Interobserver agreement for the assessment of handicap in stroke patients. Stroke.

[21] Haywood K, Whitehead L, Nadkarni VM (2018). COSCA (Core Outcome Set for Cardiac Arrest) in adults: an advisory statement from the International Liaison Committee on Resuscitation. Resuscitation.

[22] Obuchowski NA, McClish DK (1997). Sample size determination for diagnostic accuracy studies involving binormal ROC curve indices. Stat Med.

[23] DeLong ER, DeLong DM, Clarke-Pearson DL (1988). Comparing the areas under two or more correlated receiver operating characteristic curves: a nonparametric approach. Biometrics.

[24] Vickers AJ, Elkin EB (2006). Decision curve analysis: a novel method for evaluating prediction models. Med Decis Making.

[25] White IR, Royston P, Wood AM (2011). Multiple imputation using chained equations: issues and guidance for practice. Stat Med.

[26] Isenschmid C, Luescher T, Rasiah R (2019). Performance of clinical risk scores to predict mortality and neurological outcome in cardiac arrest patients. Resuscitation.

[27] Potpara TS, Mihajlovic M, Stankovic S (2017). External validation of the simple NULL-PLEASE clinical score in predicting outcome of out-of-hospital cardiac arrest. Am J Med.

[28] Simultaneous external validation of various cardiac arrest prognostic scores: a single-center retrospective study - PubMed. https://pubmed.ncbi.nlm.nih.gov/34391466/. Accessed 17 Jun 2023 10.1186/s13049-021-00935-w PMC8364702 34391466

[29] Blatter R, Amacher SA, Bohren C (2022). Comparison of different clinical risk scores to predict long-term survival and neurological outcome in adults after cardiac arrest: results from a prospective cohort study. Ann Intensive Care.

[30] Heo WY, Jung YH, Lee HY (2022). External validation of cardiac arrest-specific prognostication scores developed for early prognosis estimation after out-of-hospital cardiac arrest in a Korean multicenter cohort. PLoS ONE.

[31] Deye N, Nguyen P, Vodovar N (2020). Protein S100B as a reliable tool for early prognostication after cardiac arrest. Resuscitation.

[32] Hoiland RL, Rikhraj KJK, Thiara S (2022). Neurologic prognostication after cardiac arrest using brain biomarkers: a systematic review and meta-analysis. JAMA Neurol.

[33] Humaloja J, Lähde M, Ashton NJ (2022). GFAp and tau protein as predictors of neurological outcome after out-of-hospital cardiac arrest: a post hoc analysis of the COMACARE trial. Resuscitation.

[34] Oddo M, Sandroni C, Citerio G (2018). Quantitative versus standard pupillary light reflex for early prognostication in comatose cardiac arrest patients: an international prospective multicenter double-blinded study. Intensive Care Med.

[35] Ishikawa S, Niwano S, Imaki R (2013). Usefulness of a simple prognostication score in prediction of the prognoses of patients with out-of-hospital cardiac arrests. Int Heart J.

[36] Chen C-T, Lin J-W, Wu C-H (2022). A simple risk score for predicting neurologic outcome in out-of-hospital cardiac arrest patients after targeted temperature management. Crit Care Med.

[37] Bae DH, Lee HY, Jung YH (2021). PROLOGUE (PROgnostication using LOGistic regression model for Unselected adult cardiac arrest patients in the Early stages): development and validation of a scoring system for early prognostication in unselected adult cardiac arrest patients. Resuscitation.

[38] Wong XY, Ang YK, Li K (2022). Development and validation of the SARICA score to predict survival after return of spontaneous circulation in out of hospital cardiac arrest using an interpretable machine learning framework. Resuscitation.

[39] Lin J-J, Huang C-H, Chien Y-S (2022). TIMECARD score: an easily operated prediction model of unfavorable neurological outcomes in out-of-hospital cardiac arrest patients with targeted temperature management. J Formos Med Assoc.

[40] Shih H-M, Chen Y-C, Chen C-Y (2019). Derivation and validation of the SWAP score for very early prediction of neurologic outcome in patients with out-of-hospital cardiac arrest. Ann Emerg Med.

[41] Coppler PJ, Elmer J, Calderon L (2015). Validation of the Pittsburgh cardiac arrest category illness severity score. Resuscitation.

