# Supplementary material for: Prospective comparison of prognostic scores for prediction of outcome after out-of-hospital cardiac arrest: results of the AfterROSC1 multicentric study
Source: Ann Intensive Care. 2023 Oct 11;13:100. doi: 10.1186/s13613-023-01195-w (PMC10567621; doi:10.1186/s13613-023-01195-w)
Supplement: Supplementary file 1 — Additional file 1: Table S1. Description of each score. [file 13613_2023_1195_MOESM1_ESM.docx]

|  | History of ischemic heart disease | Age | Rhythm | Witness | Noflow duration | Lowflow duration | Location | Epinephrine | pH | Lactates | Glycemia | Brain reflexes | Glasgow score | PaCO2 | Creatinemia | Left Ventricular ejection fraction at admission | Post-ressuscitation shock | Cardiac cause |
| --- | --- | --- | --- | --- | --- | --- | --- | --- | --- | --- | --- | --- | --- | --- | --- | --- | --- | --- |
| CAHP |  | X | X |  | X | X | X | X Dose | X |  |  |  |  |  |  |  |  |  |
| sCAHP |  | X | X |  | X | | X | X Dose | X |  |  |  |  |  |  |  |  |  |
| mCAHP |  | X | X |  | X | | X | X Dose | X |  |  |  |  |  |  |  |  |  |
| OHCA |  |  | X |  | X | X |  |  |  | X |  |  |  |  | X |  |  |  |
| CREST | X |  | X |  | X | |  |  |  |  |  |  |  |  |  | X | X |  |
| C-Graph | X |  | X |  |  |  |  |  | X |  | X |  |  |  |  |  |  |  |
| TTM |  | X | X |  | X | X | X | X | X |  |  | X | X | X |  |  |  |  |
| rCAST |  |  | X | X |  |  |  |  | X | X |  |  | X |  |  |  |  |  |
| NULL-PLEASE |  | X | X | X | X | X |  |  | X | X |  |  |  |  | X |  |  | X |
| MIRACLE2 |  | X | X | X |  |  |  | X | X |  |  | X |  |  |  |  |  |  |
