# Supplementary material for: Prospective comparison of prognostic scores for prediction of outcome after out-of-hospital cardiac arrest: results of the AfterROSC1 multicentric study
Source: Ann Intensive Care. 2023 Oct 11;13:100. doi: 10.1186/s13613-023-01195-w (PMC10567621; doi:10.1186/s13613-023-01195-w)
Supplement: Supplementary file 2 — Additional file 2: Table S2. Comparison of AUC after multiple imputations. [file 13613_2023_1195_MOESM2_ESM.docx]

|  | Utstein | CAHP | sCAHP | mCAHP | OHCA | CREST | C-GRApH | TTM | NULL-PLEASE | rCAST | MIRACLE2 |
| --- | --- | --- | --- | --- | --- | --- | --- | --- | --- | --- | --- |
| Number of items for score determination | 8 | 7 | 6 | 6 | 5 | 5 | 5 | 10 | 9 | 5 | 7 |
| AUROC (95%CI) | 0.79  [0.76-0.83] | 0.87  [0.84-0.90] | 0.85  [0.81-0.88] | 0.86  [0.83-0.89] | 0.85  [0.81-0.88] | 0.79  [0.75-0.83] | 0.76  [0.71-0.80] | 0.89  [0.86-0.92] | 0.81  [0.77-0.84] | 0.82  [0.78-0.85] | 0.86  [0.82-0.89] |

eTable 1: Comparison after multiple imputations

AUROC: Area Under the ROC Curve.
