# Supplementary material for: Prospective comparison of prognostic scores for prediction of outcome after out-of-hospital cardiac arrest: results of the AfterROSC1 multicentric study
Source: Ann Intensive Care. 2023 Oct 11;13:100. doi: 10.1186/s13613-023-01195-w (PMC10567621; doi:10.1186/s13613-023-01195-w)
Supplement: Supplementary file 3 — Additional file 3: Table S3. Comparison of scores in the restricted population of patients with cardiac arrests from non-cardiac causes. [file 13613_2023_1195_MOESM3_ESM.docx]

|  | Utstein | CAHP | sCAHP | mCAHP | OHCA | CREST* | C-GRApH | TTM | NULL-PLEASE* | rCAST | MIRACLE2 |
| --- | --- | --- | --- | --- | --- | --- | --- | --- | --- | --- | --- |
| Number of items for score determination | 8 | 7 | 6 | 6 | 5 |  | 5 | 10 |  | 5 | 7 |
| Number of patients with calculated score | 233 | 230 | 230 | 230 | 226 |  | 223 | 200 |  | 224 | 222 |
| Proportion of patients with score available as compared to full cohort | 100.0% | 98.7% | 98.7% | 98.7% | 96.9% |  | 95.7% | 85.8% |  | 96.1% | 95.2% |
| Median score, IQR | NA | 159  [131-181] | 140  [117-159] | 101  [86-112] | 37  [22-46] |  | 2  [2-3] | 19  [15-22] |  | 10.5  [8.5-13] | 4.5  [4-5] |
| AUROC (95%CI) | 0.75  [0.67-0.83] | 0.85  [0.73-0.92] | 0.83  [0.76-0.90] | 0.85  [0.79-0.92] | 0.82  [0.74-0.90] |  | 0.59  [0.48-0.70] | 0.87  [0.81-0.93] |  | 0.76 [0.66-0.85] | 0.80  [0.72-0.89] |
| Hosmer-Lemeshow  - Absolute value  - P value | 8.88  0.35 | 5.79  0.67 | 4.55  0.80 | 3.92  0.86 | 12.90  0.11 |  | 1.64  0.80 | 9.94  0.26 |  | 8.28  0.40 | 2.59  0.95 |
| Comparison versus Utstein | NA | 0.01 | 0.09 | 0.01 | 0.20 |  | 0.0005 | 0.008 |  | 0.91 | 0.28 |

eTable 2: Comparison (Total sample size for non-cardiac cause, N=233)

* Not enough patients in one modality to allow prediction determination.
