# Supplementary figures and images for: Prospective comparison of prognostic scores for prediction of outcome after out-of-hospital cardiac arrest: results of the AfterROSC1 multicentric study
Source: Ann Intensive Care. 2023 Oct 11;13:100. doi: 10.1186/s13613-023-01195-w (PMC10567621; doi:10.1186/s13613-023-01195-w)

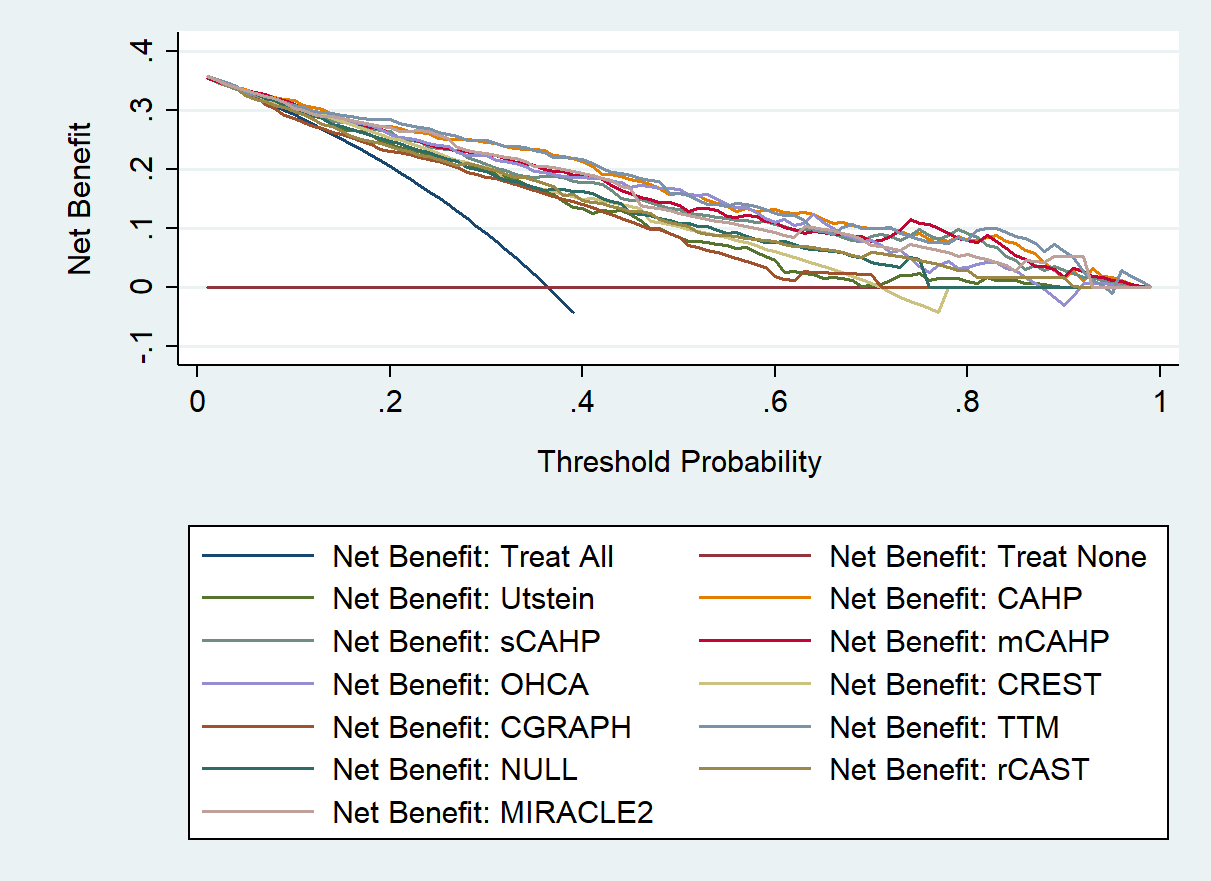

Supplement: Supplementary file 4 — Additional file 4: Figure S1. Decision curve. [file 13613_2023_1195_MOESM4_ESM.docx]
